# Hederagenin suppresses glioma cell biological activities via Nur77 in vitro study

**DOI:** 10.1002/fsn3.3163

**Published:** 2022-12-07

**Authors:** Yuxiang Dai, Ngarmbaye Masra, Lu Zhou, Chen Yu, Wei Jin, Hongbin Ni

**Affiliations:** ^1^ Department of Neurosurgery, Nanjing Drum Tower Hospital The Affiliated Hospital of Nanjing University Medical School Nanjing China

**Keywords:** AKT, glioma, Hed, Nur77, PI3K, U251, U87

## Abstract

The aim of this research was to discuss Hederagenin's antitumor effects on glioma by in vitro study. U251 and U87 cell lines were used as research target in our research. In the first step, the different Hed concentrations were treated to U251 and U87 cell lines, and the second step is Nur77 transfection in U251 and U87 with Hed treatment; measuring cell proliferation by MTT and EdU staining; evaluating cell invasion and migration abilities by transwell assay and relative gene and protein expressions by RT‐qPCR and WB assay. Compared with NC group, U251 and U87 cell proliferation were significantly depressed with cell apoptosis significantly increasing, and cell invasion and migration abilities were significantly inhibited in Hed‐treated groups (*p* < .05, respectively); however, with Nur77 transfection, the Hed's antitumor effects disappeared. Meanwhile, with Hed supplement, Nur77, PI3K, and AKT gene expressions were significantly downregulated (*p* < .05, respectively) in Hed‐treated groups; and Nur77, p‐PI3K, and p‐AKT protein expressions were significantly decreased (*p* < .05, respectively) in Hed‐treated groups. Hed had antitumor effects on glioma cell biological activities via Nur77/PI3K/AKT pathway in vitro study.

## Graphical abstract

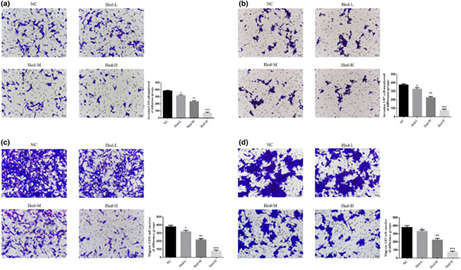

## 1 INTRODUCTION

Glioma is a common malignant brain tumor in the nervous system; proportions taken by it in intracranial tumors and malignant intracranial tumors are known to be 45% and 80%, respectively. In terms of incidence and mortality, glioma ranks first among malignant central nervous system (CNS) neoplasms (Tsang et al., 1993). Glioma cells show infiltratively growth; and, the boundary between these cells and normal brain tissues is still not clear. In addition, these cells are also featured with high incidence, short disease course, high morbidity, a high recurrence rate, and a low cure rate (Hamada et al., 1996). At present, the major glioma treatment approach is a combination of surgical operation and chemoradiotherapy. Although symptoms of patients can, thus, be improved in a short time, it is apt to recur due to residual diseases; and, the corresponding prognosis is also rather poor (Li et al., 2016). In recent years, a variety of natural products have shown good effects in the prevention and treatment of glioma (Cao et al., 2017; Lin et al., 2021; Park et al., 2018). Hederagenin (Hed) belongs to triterpenoid acids, which are abundant in ivy leaves and have a wide range of biological activities (Rodríguez‐Hernández et al., 2015). Relative studies found that Hed had antitumor effects in NSCLC, colon cancer, and leukemia in previous research (Chen et al., 2019; Liu et al., 2014; Mimaki et al., 1999). Our present study firstly discussed Hed's antitumor effects on glioma and observed Hed depression on cell proliferation, apoptosis, invasion, and migration abilities in glioma cell lines (U87 and U251) and relative mechanisms by in vitro study.

## 2 MATERIALS AND METHODS

### 2.1 Cell and reagent

Heb was purchased from Sigma (cat.no. H3916; USA); U251 and U87 cell lines were from the Cell bank of typical culture collection Committee of Chinese Academy of Sciences. Medium and fetal bovine serum – BI, USA; MTT kit – Sigma, USA; EdU kit – Keygen, Nanjing, China; BCA protein concentration kit – Keygen, Nanjing, China; antibodies including Nur77, PI3K, AKT, p‐AKT, p‐PI3K, and GAPDH – Abcam, USA.

### 2.2 Cell culture

Cells were routinely cultured in DMEM containing 10% fetal bovine serum at 37°C and 5%CO_2_; moreover, the humidity is saturated. The culture medium was changed every other day and passage was conducted once every 3 days. Once cell growth was completed by 60–70% and confluence took place, the serum concentration was lowered to 5%; afterward, 1‐μg plasmids were mixed with 100 μl pcDNA3.1 for 20 min and their mixture was added into the medium. Four hours later, the preceding medium was replaced with a 20% serum‐containing medium in which cell culturing proceeds. Moreover, Nur77 was designed in Jiangsu KeyGEN BioTECH Co., Ltd.

### 2.3 Cells grouping

NC: the cells (U251 and U87) were treated with normal; Heb‐L: the cells (U251 and U87) were treated with 5 μM Hed; Hed‐M: the cells (U251 and U87) were treated with 10 μM Hed; Hed‐H: the cells (U251 and U87) were treated with 20 μM Hed; pcDNA3.1: the cells (U251 and U87) were transfected with pcDNA3.1; Hed: the cells (U251 and U87) were treated with 20 μM Hed; Hed + Nur77: the cells (U251 and U87) transfected with Nur77 by pcDNA3.1 and were treated with 20 μM Hed.

### 2.4 MTT assay

After 0 h, 24 h, 48 h, and 72 h of cell treatment, 20 μl MTT (5 mg/ml) was added into each well, then the well was incubated and removed after 4 h. The liquid in the 96‐well plate was removed using suction and 150‐μl DMSO was added to each well. Subsequent to a 15‐min reaction at room temperature, the plate was placed in a microplate reader to determine the absorbance value at a wavelength of 490 nm. Each experiment was repeated in triplicate.

### 2.5 EdU assay

After 48 h of cell treatment, using EdU infiltrate working fluid to incubate at 37°C for 3 h, 4% paraformaldehyde fixation for 30 min, after neutralization of excess paraformaldehyde with glycine, adding 0.5%Trition X‐100 to incubate 10 min, washing by PBS, adding dye solution to incubate at 37°C for 30 min, wash off excess staining solution with PBS, Nuclei were stained with DAPI for 3 min, pictures were taken under the fluorescence microscope. Blue is the nucleus, and green is the EdU‐positive cells, that is, newly proliferated cells. Randomly select five fields to obtain the average value under 200‐fold, counting EdU‐positive cell number to reflect the cell proliferation ability.

### 2.6 Annexin V‐FITC/PI double staining apoptosis analysis

After 48 h of cell treatment, the cells were collected and rinsed with PBS according to the instructions provided with the Annexin V‐FITC Apoptosis Detection Kit. A flow cytometer was used to determine the rate of apoptosis. Each experiment was repeated in triplicate.

### 2.7 Transwell assay to invasion

After blending Matrigel with DMEM at a ratio of 1:2 on ice, the mixture was added into the transwell cabin (30 μl in each well). Together with a 24‐well plate, the transwell cabin was placed in an incubator for 1 hour and then taken out to remove the nonsolidified Matrigel using suction. 100 μl of cells (treated and cultured using various methods) and 100 μl of serum‐free DMEM were added to the upper cabin. A serum‐containing DMEM corresponding to the concentration of a drug was added to the lower cabin. Next, the transwell cabins were placed in an incubator to culture for 24 h and then removed. The medium was abandoned and the cells were fixed for 10 minutes using 4% paraformaldehyde, any cells failing to penetrate through the Matrigel were wiped away using swabs. After staining using 0.1% crystal violet, the excess crystal violet was rinsed away using PBS. Once dried, photographs were taken by optical microscope (CX23, Olympus, Japan). The cells were counted using the photographs of each group. Each experiment was repeated in triplicate.

### 2.8 Transwell assay to migration

Inoculated cells, adjust the cell density to 1 × 10^5^cell/ml, take 100 μl of cell suspension and add it into the Transwell chamber, and add 500 μl of FBS‐containing medium into the lower chamber; The 24‐well cell culture plate was placed in a 5% CO_2_ incubator at 37°C for 24 h; Wipe the Matrigel and the cells in the upper chamber with a cotton swab, remove the Transwell, invert, air dry, add 500 μl of 0.1% crystal violet into the 24‐well plate, place the chamber in it, immerse the membrane in the dye, take it out after 30 min at 37°C, clean it with PBS, take three fields of view on the diameter, and take photos (magnification: 200×), Counting.

### 2.9 Real‐time polymerase chain reaction (RT‐qPCR) assay

To extract the total RNA, cells were added to TRIzol reagent to perform pyrolysis. A 10‐μl cDNA reaction system (reaction conditions: 42°C for 60 min, 70°C for 10 min) and a 20‐μl qRT‐PCR reaction system (reaction conditions: 90°C for 15 min, 95°C for 2 min, 95°C for 5 s, 60°C for 25 s, and a cycle of 65–95–65°C for 30 min) were both prepared. After 40 circulations, fluorescence detection was performed. Here, the relative expression of the gene was determined using the delta–delta CT method. Each experiment was repeated in triplicate. The sequence as follows: PI3K: F: 5′‐TATTTGGACTTTGCGACAAGACT‐3′ and R: 5′‐TCGAACGTACTGGTCTGGATAG‐3′; AKT: F: 5′‐AGCGACGTGGCTATTGTGAAG‐3′ and 5′‐GCCATCATTCTTGAGGAGGAAGT‐3′; GAPDH: F: 5′‐GATTCCCTGGACCTAAAGGTGC‐3′ and R: 5′‐AGCCTCTCCATCTTTGCCAGCA‐3′; Nur77: F: 5′‐TCATGGACGGCTACAGAG‐3′; R: 5′‐GTAGGCATGGAATAGCTC‐3′.

### 2.10 Western blotting (WB) assay

After 48 h of cell treatment, a protein lysis buffer was used to extract the total protein and the protein concentration was determined using a BCA kit (CoWin Biosciences, Beijing). Following the addition of the same amount of protein, polyacrylamide gel electrophoresis was implemented using 100 g/L sodium lauryl sulfate. Once the electrophoresis was completed, the protein was transferred onto a 0.45 μm PVDF membrane, which was then sealed in a confining liquid. The phosphorylated and nonphosphorylated proteins were sealed using 5% fetal bovine serum and 50 g/L skimmed milk powder, respectively. After sealing, the primary and secondary antibodies were added accordingly. This point represents the completion of the method development. The primary antibodies used here were as follows: rabbit anti‐GAPDH (1:5000, Affinity, USA); rabbit anti‐Nur77 (1:1000, Proteintech, USA); and rabbit anti‐CXCR4, rabbit anti‐PI3K and p‐PI3K, AKT, and p‐AKT (all 1:1000, Abcam, UK). The secondary antibody used here was goat anti‐rabbit IgG (1:1000, CST, USA). Each experiment was repeated in triplicate.

### 2.11 Statistical analysis

The relevant statistical analyses were performed using SPSS 22.0. The corresponding data were expressed as the mean ± standard deviation (mean ± SD), where the t‐test was applied. It was found that the value of *p* was below 0.05 (*p* < .05), indicating that there are statistically significant differences between the data.

## 3 RESULTS

### 3.1 Hed affected cell proliferation and EdU‐positive cell number

**FIGURE 1:**
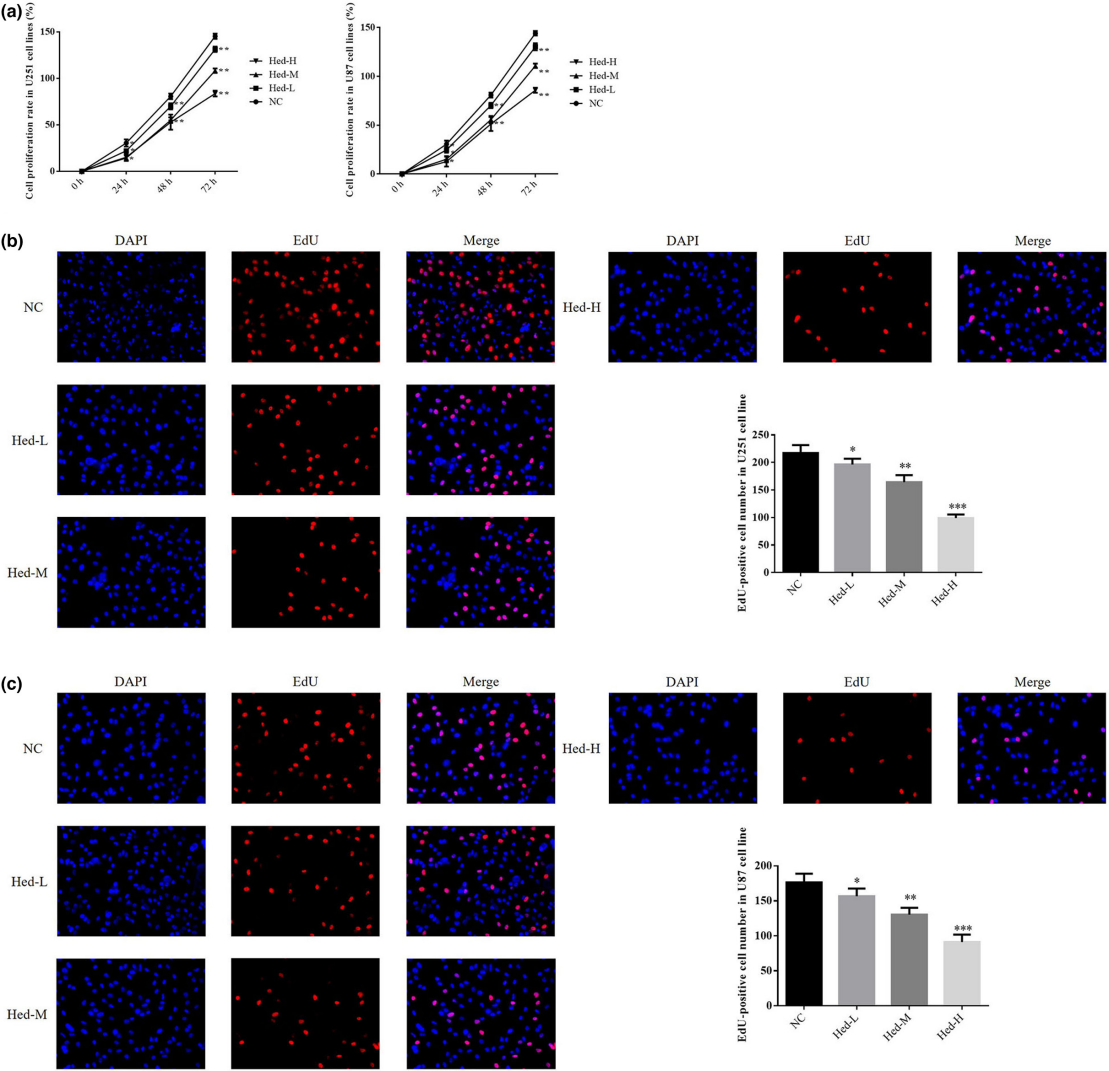
Hed affected cell proliferation and EdU‐positive cell number. NC: the cells (U251 and U87) were treated normal; Heb‐L: the cells (U251 and U87) were treated with 5 μM Hed; Hed‐M: the cells (U251 and U87) were treated with 10 μM Hed; Hed‐H: the cells (U251 and U87) were treated with 20 μM Hed. Cell proliferation rate in different cell lines (%). EdU‐positive cell number in U251 cell line (×200). EdU‐positive cell number in U87 cell line (×200). *p < .05, **p < .01, ***p < .001, compared with NC group.

By MTT assay, compared with NC group, the cell proliferation rate of U251 and U87 cell lines was significantly depressed at 24 h, 48 h, and 72 h in Hed‐L, Hed‐M, and Hed‐H groups (*p* < .05, *p* < .01, or *p* < .001, respectively, Figure 1a). By EdU assay, compared with NC group, EdU‐positive cell number of U251 and U87 cell lines was significantly reduced in Hed‐L, Hed‐M, and Hed‐H groups (*p* < .001, respectively, Figure 1b,c).

### 3.2 Hed affects cell apoptosis rate

**FIGURE 2:**
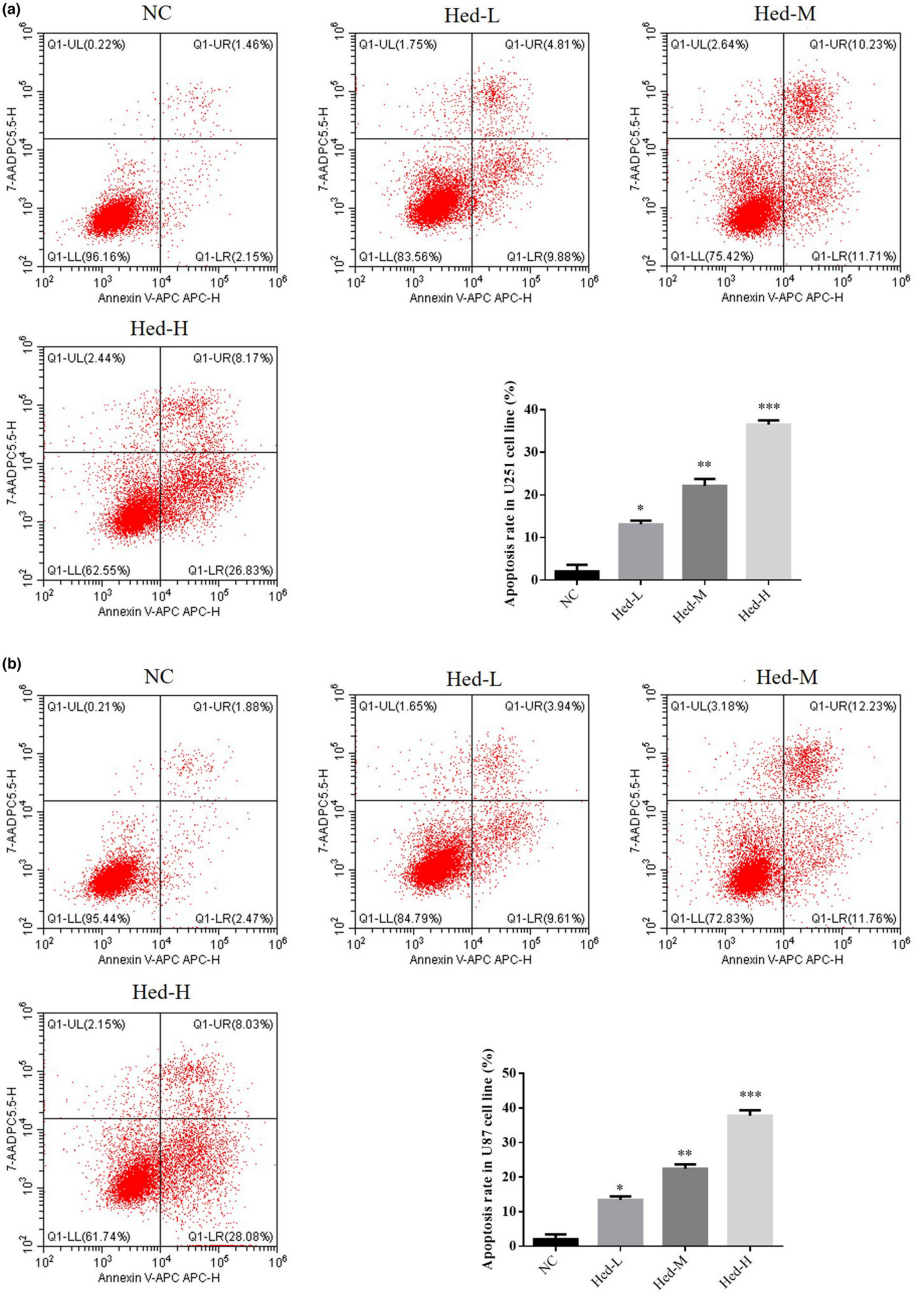
Hed affects cell apoptosis rate. NC: the cells (U251 and U87) were treated normal; Heb‐L: the cells (U251 and U87) were treated with 5 μM Hed; Hed‐M: the cells (U251 and U87) were treated with 10 μM Hed; Hed‐H: the cells (U251 and U87) were treated with 20 μM Hed. Apoptosis rate in U251 cell lines (%). Apoptosis rate in U87 cell lines (%). *p < .05, **p < .01, ***p < .001, compared with NC group.

By flow cytometry (Annexin V‐FITC/PI double staining apoptosis analysis), compared with NC group, apoptosis cell rate of Hed‐L, Hed‐M, and Hed‐H groups was significantly upregulated in U251 and U87 cell lines (*p* < .05, *p* < .01, or *p* < .001, respectively, Figure 2a,b).

### 3.3 Hed affects cell invasion and migration ability

**FIGURE 3:**
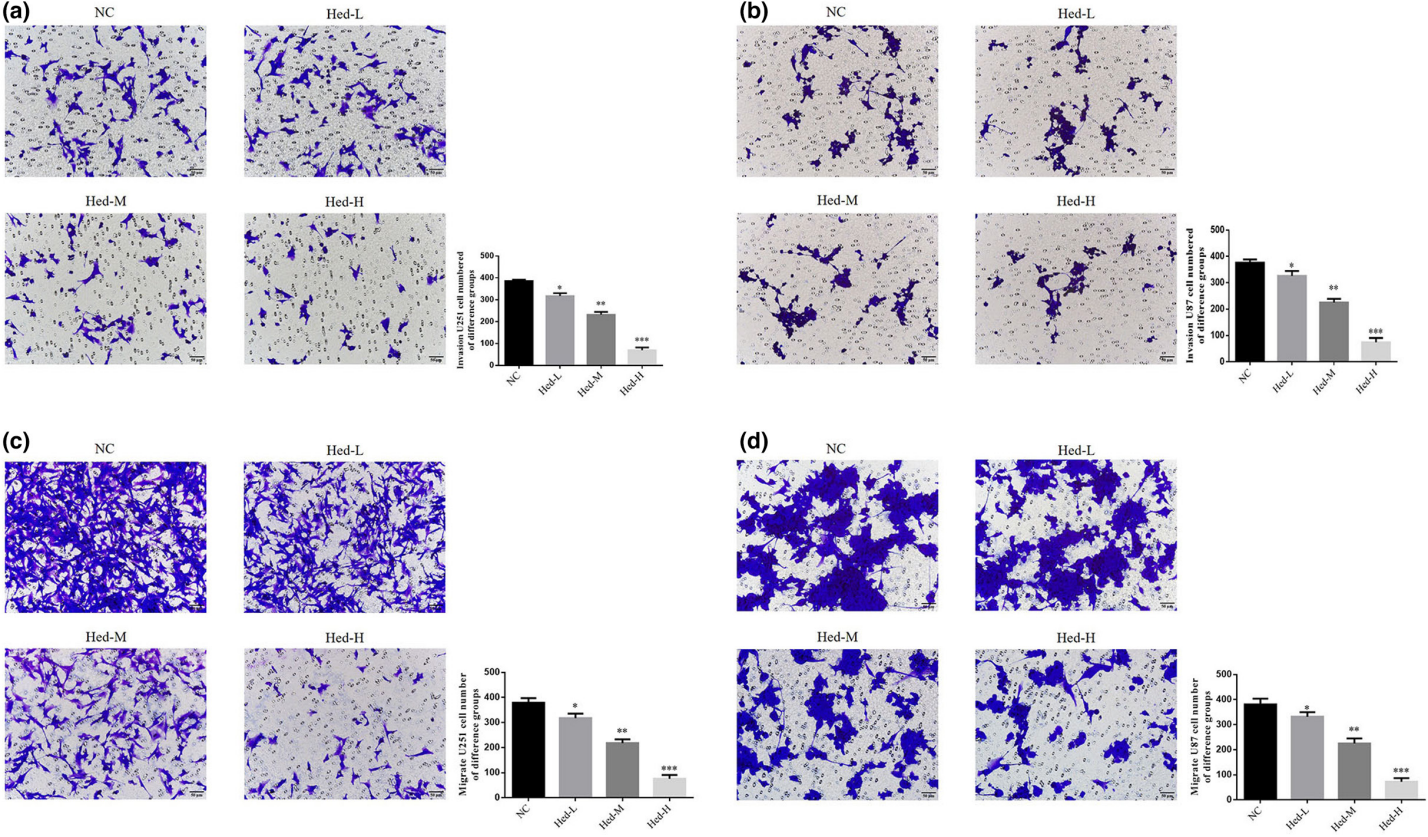
Hed affects cell invasion and migration ability. NC: the cells (U251 and U87) were treated normal; Heb‐L: the cells (U251 and U87) were treated with 5 μM Hed; Hed‐M: the cells (U251 and U87) were treated with 10 μM Hed; Hed‐H: the cells (U251 and U87) were treated with 20 μM Hed. U251 cell number invasion in different groups (×200). U87 cell number invasion in different groups (×200). U251 cell number migration in different groups (×200). U87 cell number migration in different groups (×200). *p < .05, **p < .01, ***p < .001, compared with NC group.

By transwell assay to observe cell invasion and migration abilities, compared with NC group, the invasion and migration cell number of Heb‐L, Hed‐M, and Hed‐H groups were significantly depressed in U251 and U87 cell lines (*p* < .05, *p* < .01, or *p* < .001, respectively, Figure 3a,d).

### 3.4 Hed affects relative mRNA expressions

**FIGURE 4:**
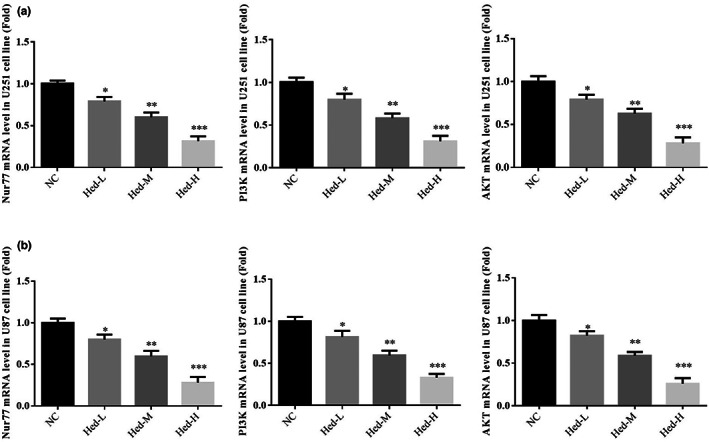
Hed affects relative mRNA expressions. NC: the cells (U251 and U87) were treated normal; Heb‐L: the cells (U251 and U87) were treated with 5 μM Hed; Hed‐M: the cells (U251 and U87) were treated with 10 μM Hed; Hed‐H: the cells (U251 and U87) were treated with 20 μM Hed. Hed affects relative mRNA expression in U251 cell line. Hed affects relative mRNA expression in U87 cell line. *p < .05, **p < .01, ***p < .001, compared with NC group.

By RT‐qPCR assay, compared with NC group, Nur77, PI3K, and AKT mRNA expressions of Hed‐L, Hed‐M, and Hed‐H groups were significantly downregulated in U251 and U87 cell lines (*p* < .05, *p* < .01, or *p* < .001, respectively, Figure 4a,b).

### 3.5 Hed affects relative protein expressions by WB assay

**FIGURE 5:**
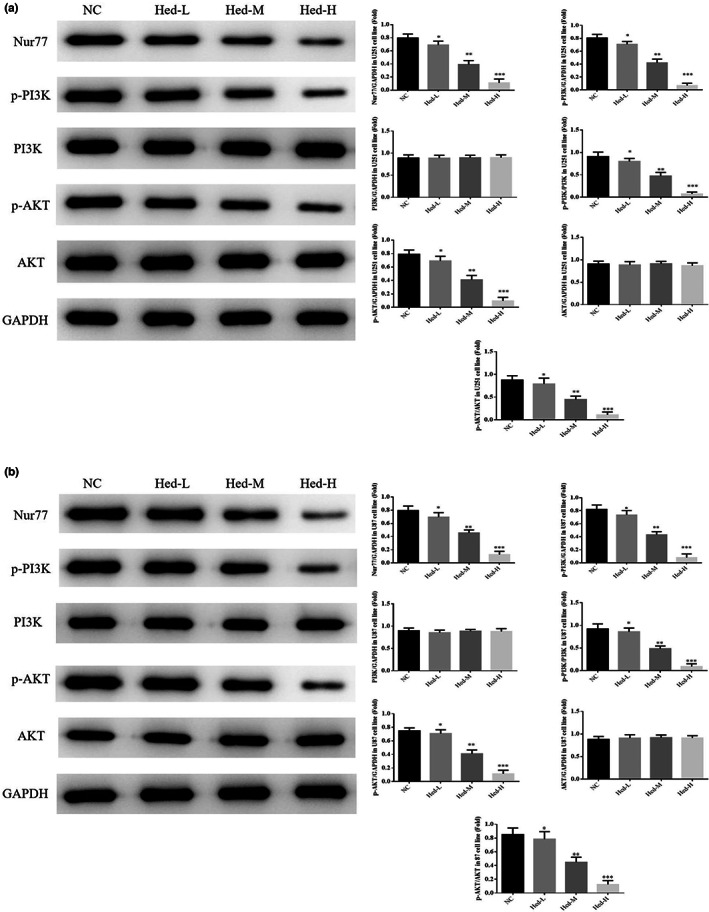
Hed affects relative protein expressions by WB assay. NC: the cells (U251 and U87) were treated normal; Heb‐L: the cells (U251 and U87) were treated with 5 μM Hed; Hed‐M: the cells (U251 and U87) were treated with 10 μM Hed; Hed‐H: the cells (U251 and U87) were treated with 20 μM Hed. Hed affects relative protein expressions by WB assay in U251 cell line. Hed affects relative protein expressions by WB assay in U87 cell line. *p < .05, **p < .01, ***p < .001, compared with NC group.

By WB assay, PI3K and AKT protein expressions had no significant differences among NC, Hed‐L, Hed‐M, and Hed‐H groups in U251 and U87 cell lines (*p* > .05, respectively, Figure 5a,b); however, compared with NC group, Nur77, p‐PI3K, p‐AKT, p‐PI3K/PI3K, and p‐AKT/AKT were significantly different in Hed‐L, Hed‐M, and Hed‐H groups in U251 and U87 cell lines (*p* < .05, *p* < .01, or *p* < .001, respectively, Figure 5a,b).

### 3.6 Nur77's effect on Hed's antitumor effects in cell proliferation and EdU‐positive cell number

**FIGURE 6:**
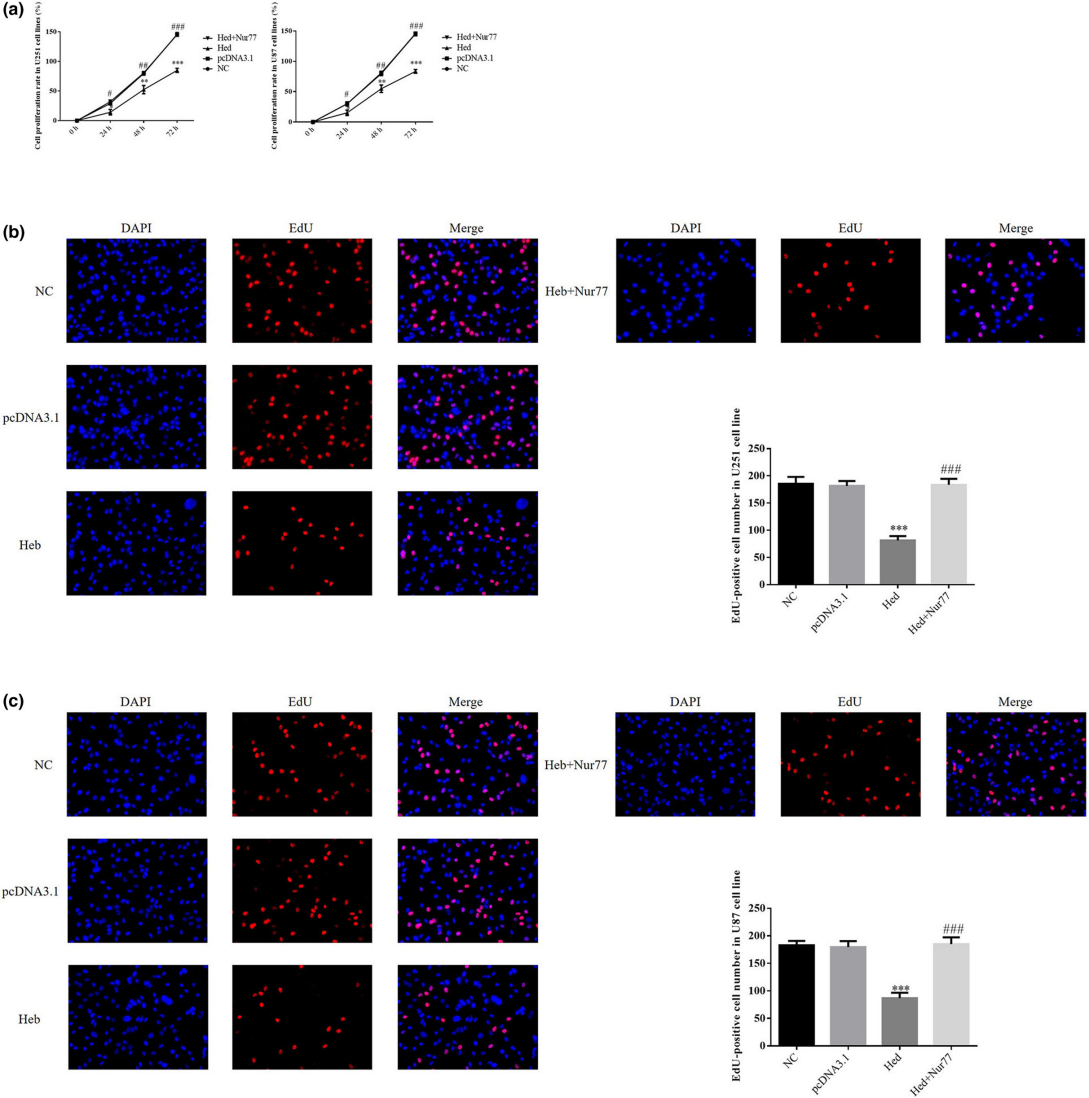
Nur77's effect of Hed's antitumor effects on cell proliferation and EdU‐positive cell number. NC: the cells (U251 and U87) were treated normal; pcDNA3.1: the cells (U251 and U87) were transfected with pcDNA3.1; Hed: the cells (U251 and U87) were treated with 20 μM Hed; Hed+Nur77: the cells (U251 and U87) transfected with Nur77 by pcDNA3.1 and were treated with 20 μM Hed. Nur77's effect of Hed's antitumor effects on cell proliferation. Nur77's effect of Hed's antitumor effects on EdU‐positive U251 cell number. Nur77's effect of Hed's antitumor effects on EdU‐positive U87 cell number. ***p < .001; compared with NC group; ###*p* < .001, compared with Hed group.

Compared with NC group, cell proliferation rates of Hed groups in 24 h, 48 h, and 72 h were significantly depressed in U251 and U87 cell lines (*p* < .05, *p* < .01, or *p* < .001, respectively, Figure 6a); however, with Nur77 supplement, compared with Hed group, cell proliferation rates of Heb+Nur 77 groups in 24 h, 48 h, and 72 h were significantly increased in U251 and U87 cell lines (*p* < .05, *p* < .01, or *p* < .001, respectively, Figure 6a). By EdU assay, compared with NC group, EdU‐positive cell number of Hed groups was significantly decreased in U251 and U87 cell lines (*p* < .001, respectively, Figure 6b); with Nur 77 transfected, EdU‐positive cell number of Hed+Nur 77 groups was significantly increased in U251 and U87 cell lines compared with Hed groups (*p* < .001, respectively, Figure 6b).

### 3.7 Nur77's effect on Hed's antitumor effects in cell apoptosis

**FIGURE 7:**
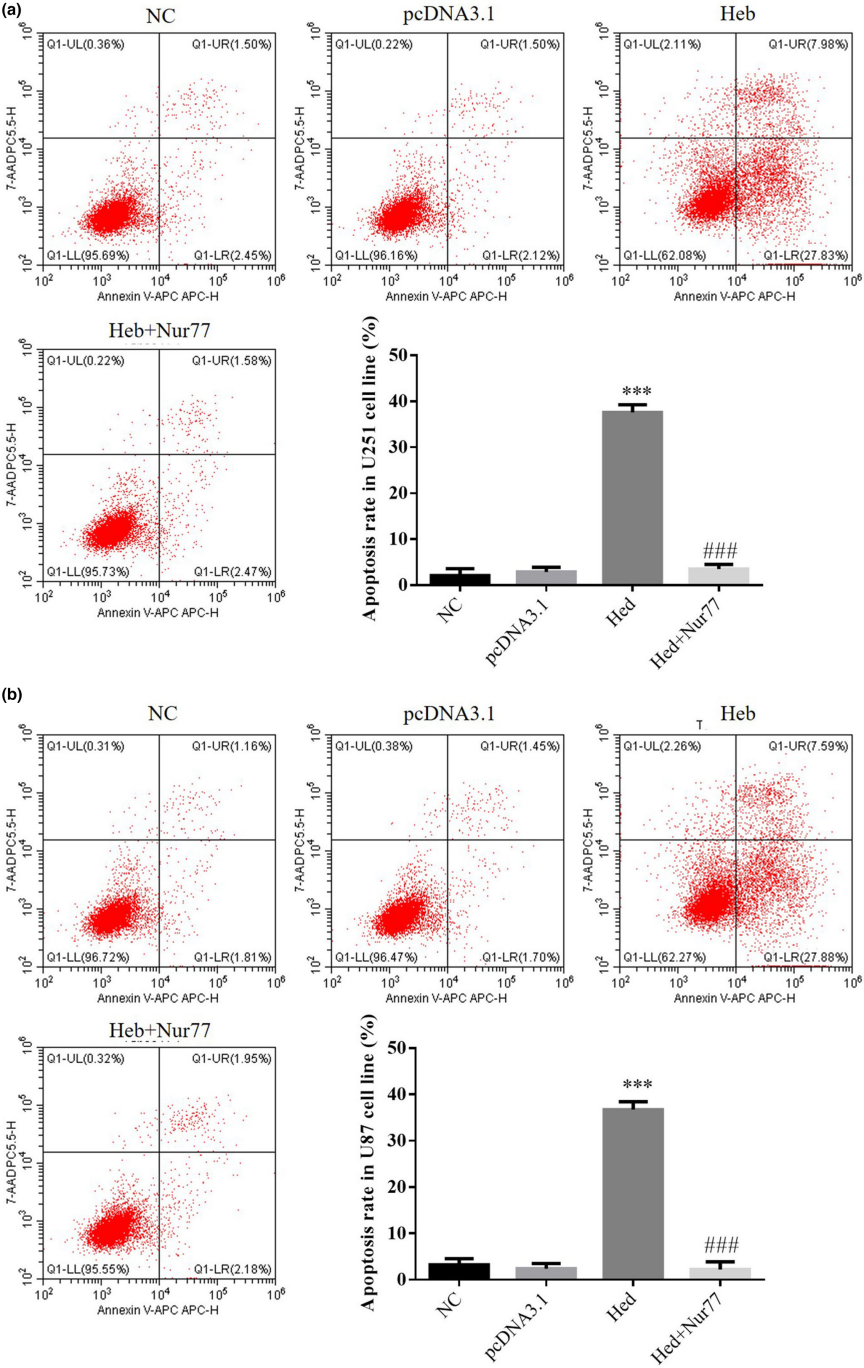
Nur77's effect of Hed's antitumor effects on cell apoptosis. NC: the cells (U251 and U87) were treated normal; pcDNA3.1: the cells (U251 and U87) were transfected with pcDNA3.1; Hed: the cells (U251 and U87) were treated with 20 μM Hed; Hed+Nur77: the cells (U251 and U87) transfected with Nur77 by pcDNA3.1 and were treated with 20 μM Hed. Nur77's effect of Hed's antitumor effects on cell apoptosis in U251 cell line. Nur77's effect of Hed's antitumor effects on cell apoptosis in U87 cell line. ***p < .001; compared with NC group; ###*p* < .001, compared with Hed group.

Compared with NC group, the cell apoptosis rate of Hed groups was significantly upregulated in U251 and U87 cell lines (*p* < .001, respectively, Figure 7a,b); with Nur 77 supplement, compared with Hed group, the cell apoptosis rate of Hed+Nur 77 groups was significantly downregulated in U251 and U87 cell lines (*p* < .001, respectively, Figure 7a,b).

### 3.8 Nur77's effect of Hed's antitumor effects on cell invasion and migration abilities

**FIGURE 8:**
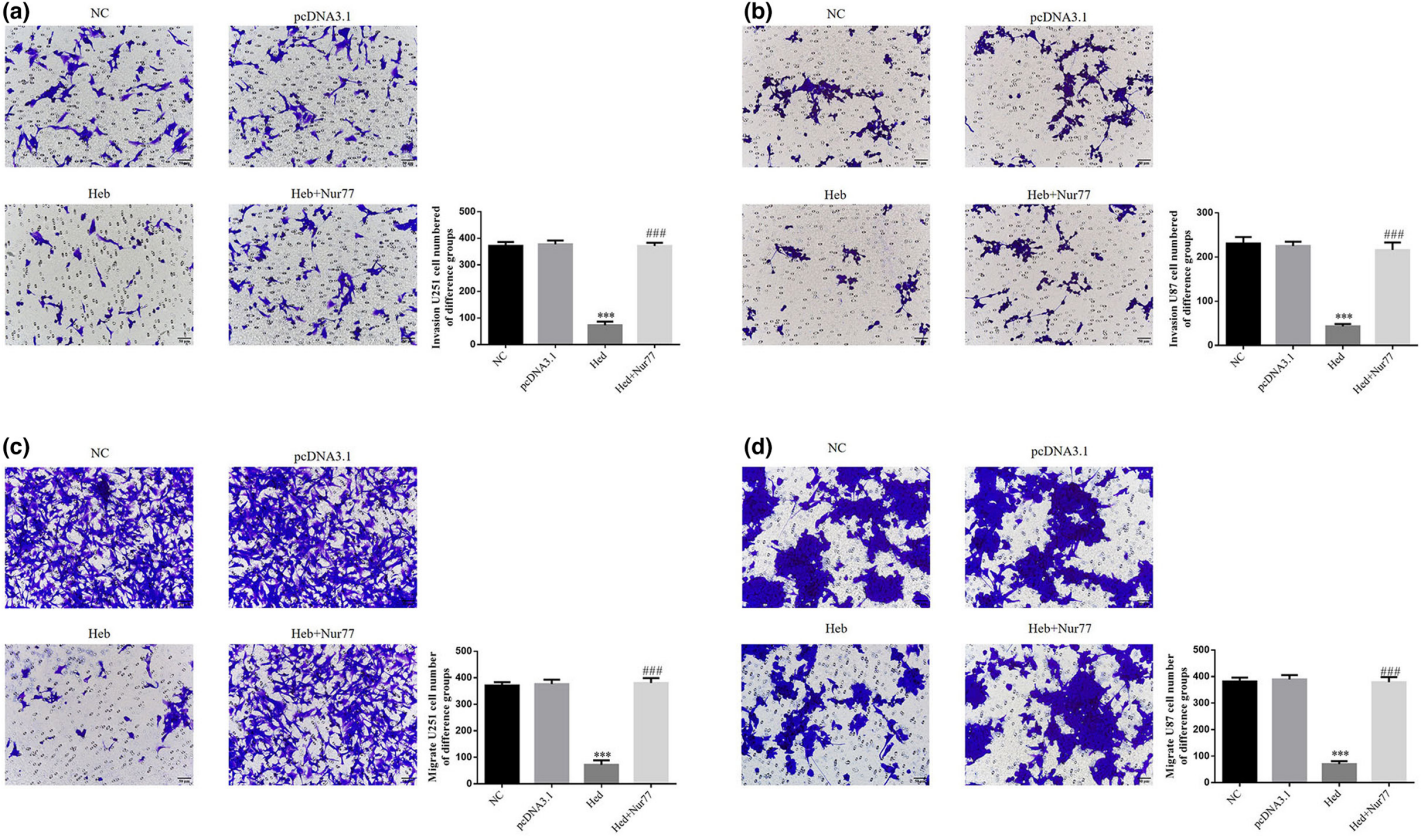
Nur77's effect of Hed's antitumor effects on cell invasion and migration abilities. NC: the cells (U251 and U87) were treated normal; pcDNA3.1: the cells (U251 and U87) were transfected with pcDNA3.1; Hed: the cells (U251 and U87) were treated with 20 μM Hed; Hed+Nur77: the cells (U251 and U87) transfected with Nur77 by pcDNA3.1 and were treated with 20 μM Hed. Nur77's effect of Hed's antitumor effects on U251 cell invasion ability (×200). Nur77's effect of Hed's antitumor effects on U287 cell invasion ability (×200). Nur77's effect of Hed's antitumor effects on U251 cell migration ability (×200). Nur77's effect of Hed's antitumor effects on U287 cell migration ability (×200). ***p < .001; compared with NC group; ###*p* < .001, compared with Hed group.

Using transwell assay to detect cell invasion and migration, compared with NC group, invasion and migration cell number of Heb groups were significantly suppressed in U251 and U87 cell lines (*p* < .001, respectively, Figure 8a–d); with Nur 77 transfection, compared with Hed group, invasion and migration cell number of Hed+Nur 77 groups were significantly enhanced in U251 and U87 cell lines (*p* < .001, respectively, Figure 8a–d).

### 3.9 Nur77, PI3K, and AKT mRNA expressions

**FIGURE 9:**
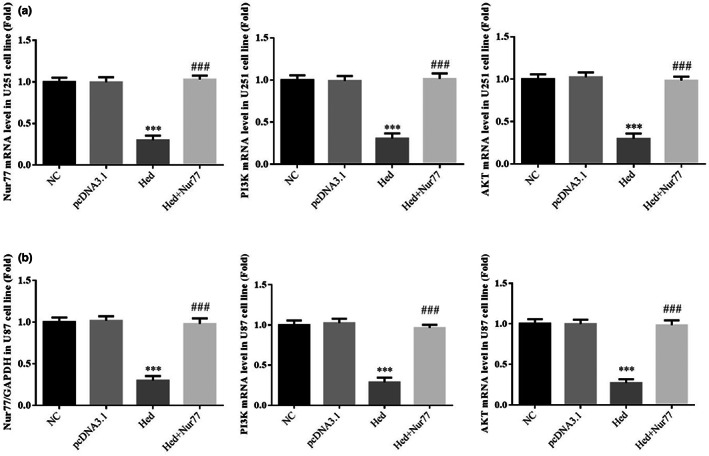
Nur77, PI3K, and AKT mRNA expressions. NC: the cells (U251 and U87) were treated normal; pcDNA3.1: the cells (U251 and U87) were transfected with pcDNA3.1; Hed: the cells (U251 and U87) were treated with 20 μM Hed; Hed+Nur77: the cells (U251 and U87) transfected with Nur77 by pcDNA3.1 and were treated with 20 μM Hed. Nur77, PI3K, and AKT mRNA expressions in U251 cell line. Nur77, PI3K, and AKT mRNA expressions in U87 cell line. ***p < .001; compared with NC group; ###*p* < .001, compared with Hed group.

By RT‐qPCR assay, compared with NC group, Nur77, PI3K, and AKT mRNA expressions of Hed groups were significantly depressed in U251 and U87 cell lines (*p* < .001, respectively, Figure 9a,b); with Nur77 supplement, compared with Hed group, Nur77, PI3K, and AKT mRNA expressions of Hed+Nur77 groups were significantly increased in U251 and U87 cell lines (*p* < .001, respectively, Figure 9a,b).

### 3.10 Nur77, PI3K, p‐PI3K, AKT, and p‐AKT protein expressions

**FIGURE 10:**
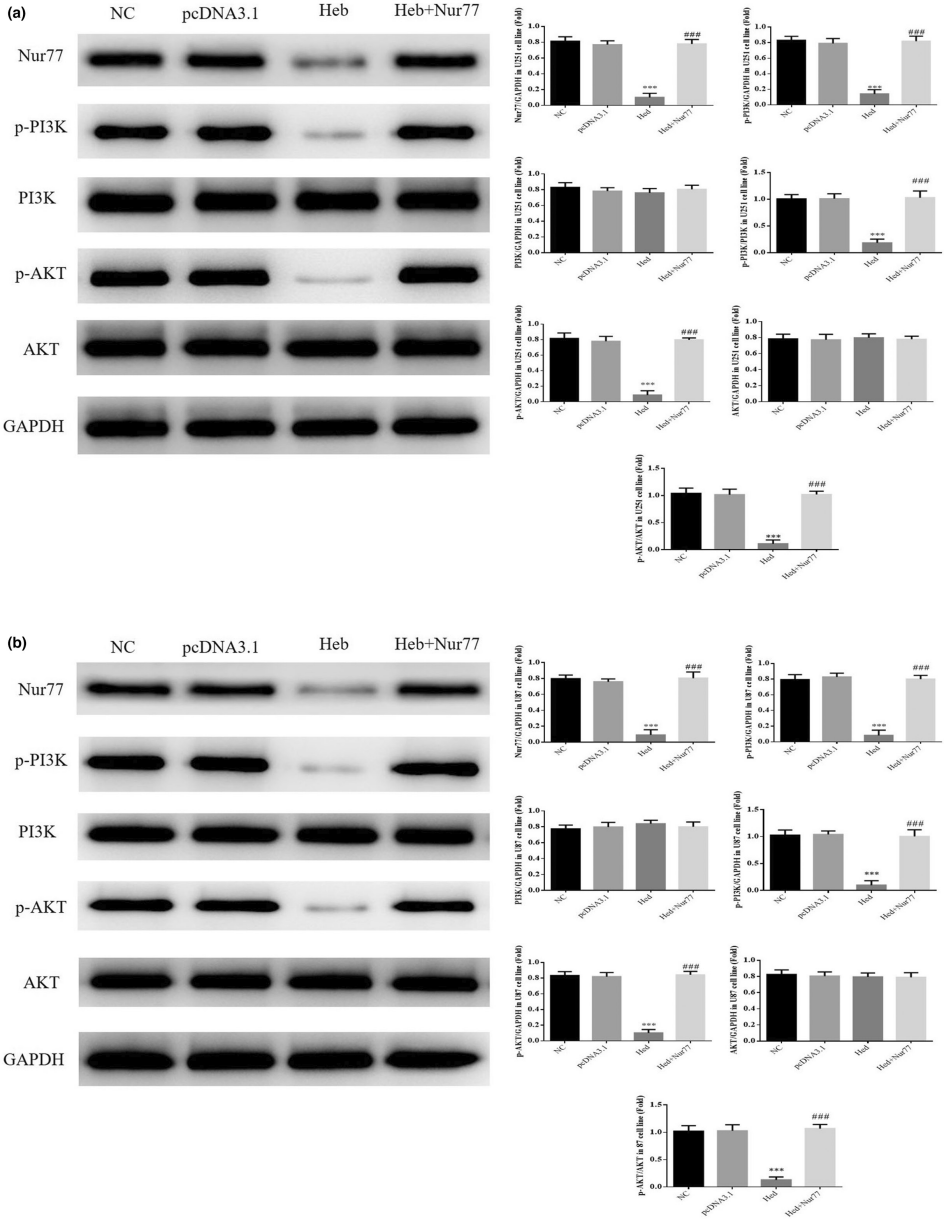
Nur77, PI3K, p‐PI3K, AKT, and p‐AKT protein expressions. NC: the cells (U251 and U87) were treated normal; pcDNA3.1: the cells (U251 and U87) were transfected with pcDNA3.1; Hed: the cells (U251 and U87) were treated with 20 μM Hed; Hed+Nur77: the cells (U251 and U87) transfected with Nur77 by pcDNA3.1 and were treated with 20 μM Hed. Nur77, PI3K, p‐PI3K, AKT, and p‐AKT protein expressions in U251 cell line. Nur77, PI3K, p‐PI3K, AKT, and p‐AKT protein expressions in U87 cell line. ***p < .001; compared with NC group; ###*p* < .001, compared with Hed group.

By WB assay, compared with NC group, Nur77, p‐PI3K, p‐AKT, p‐PI3K/PI3K, and p‐AKT/AKT of Hed groups were significantly depressed in U251 and U87 cell lines (*p*<.001, respectively, Figure 10a,b); with Nur77 supplement, compared with Hed group, Nur77, p‐PI3K, p‐AKT, p‐PI3K/PI3K, and p‐AKT/AKT of Hed+Nur77 groups were significantly improved in U251 and U87 cell lines (*p* < .001, respectively, Figure 10a,b).

## 4 DISCUSSION

The research and development of natural products have always been an important means to find new drugs and fight against cancer. The structure leading, modification, and transformation of natural products have also promoted the rapid development of antitumor drugs. Paclitaxel and irinotecan (camptothecin derivative) are widely used in clinics (Basade & Mane, 2021; Shi & Sun, 2017). In addition, a variety of anticancer substances exist in vegetables, fruits, edible fungi, spices, and other foods, which are beneficial to the prevention and treatment of human cancer. Like as, EGCG, the main component of green tea polyphenols, and resveratrol, a polyphenol compound extracted from grapes, all showed significant antitumor activity (Rauf et al., 2018; Romano & Martel, 2021).

Hed is a triterpene acid compound extracted from ivy leaves, which also exists in the leaves of Cyclocarya paliurus and Ailanthus vulgaris (Gao et al., 2016; Liu et al., 2014; Zhang et al., 2012). Research (15) found that Hed was the main active ingredient in the leaves of Cyclocarya paliurus, which inhibits the proliferation of non‐small‐cell lung cancer cell line A549. Hed could significantly inhibit the cell viability of large cell lung cancer cell NCI‐H460, colon cancer cell HT‐29, and leukemia cell CEM (Zhang et al., 2012). Further studies (Liu et al., 2014) found that Ivy saponin induces apoptosis of colon cancer cell LoVo by regulating mitochondrial pathway. The present study found that Hed had effects to suppress glioma cell activities including depressing cell proliferation, invasion, and migration. In order to discuss the clear mechanism, the results found that Hed could inhibit Nur77 mRNA and protein expression in U251 and U87 cell lines; however, with Nur77 transfection in U251 and U87, Hed's antitumor effects disappeared. Depending on these results, we inferred that Hed's antitumor effects were closely correlated with Nur77.

Nur77, also known as NR4A1, TR3, or NGF‐IB, is a member of the steroid/thyroid hormone receptor superfamily. As a transcription factor and early response gene, Nur77 in different types of cells and tissues can be induced by many irritants, including serum, inflammatory factors, growth factors, and pressure (Winoto & Littman, 2002). At present, there are still disputes about the role of Nur77 in tumors (Lee et al., 2010; Liu et al., 1994; Wu et al., 2011). In our present study, the results suggested that Hed had anticancer effects to depress Nur77, and the data also found that Nur77 was a key role in Hed's antitumor effects in glioma. Some research also found that Nur77 could target PI3K/AKT activities (Bai et al., 2015; Han et al., 2006; Huang et al., 2016; Shi et al., 2021). PI3K/AKT, an important signaling pathway in cells, gains control over multiple biological processes of cells, such as their proliferation, growth, apoptosis, transcription, translation, cytoskeletal rearrangement, and cell cycles. In addition, it also plays a crucial role in tumor occurrence and development (Deng et al., 2020; Xue et al., 2020). As far as our research findings are concerned, with Nur77 depressing, p‐PI3K and p‐AKT protein expressions and p‐PI3K/PI3Kand p‐AKT/AKT rates were significantly depressed, which might be correlated Hed's antitumor mechanism.

In conclusion, Hed had effects to depress glioma cell biological activities in vitro study. Hed suppressed glioma cell biological activities via depressing Nur77, meanwhile, Nur77 downstream which was PI3K/AKT signaling pathway was also inhibiting in our in vitro study.

## FUNDING INFORMATION

None.

## CONFLICT OF INTEREST

The authors declare that they have no competing interests.

## Data Availability

The datasets used and/or analyzed during the current study are available from the corresponding author (Ni Hongbin) whose e‐mail address is nihongbin0429@163.com.
